# A KHz frequency cold atmospheric pressure argon plasma jet for the emission of O(^1^S) auroral lines in ambient air

**DOI:** 10.1038/s41598-021-81488-x

**Published:** 2021-01-21

**Authors:** S. Jaiswal, E. M. Aguirre, G. Veda Prakash

**Affiliations:** 1 Department of Physics, Auburn University, Auburn, AL 36832 USA; 2 Centre for Energy Studies, Indian Institute of Technology Delhi, New Delhi, 110016 India; 3 Present Address: Department of Chemical and Biological Engineering, Princeton University, Princeton, NJ 08544 USA

**Keywords:** Plasma physics, Aurora, Chemical physics

## Abstract

Creation of the “auroral” green line, a fascinating occurrence commonly observed in the upper atmosphere, has long been a difficult endeavor, especially at atmospheric pressure. Here we report strong emission of the “auroral” green line for the first time in a kHz frequency, linear field atmospheric pressure plasma jet system. The device used 99.999% pure argon as a working gas for the plasma generation. Optical emission spectroscopy measurements of the after discharge region show the existence of 557.7 nm emission which corresponds to the transition O($^1$S)–O($^1$D). The intensity of the produced green line is strong enough that the entire plasma plume in the ambient air is visible as a green plasma. We provide the chemical reactions of O($^1$S) production in the plasma and the estimation of the density of the O($^1$S) metastable state using the kinetic reactions. Further, the O($^1$S) emission is characterized by changing the flow rate of argon, applied voltage and electrode gap. The adequate plasma length ($> 25 \, \hbox {mm}$) along with the production of a variety of reactive components viz; OH, ${\hbox {N}}_{2}^{+}$ and oxygen (777 nm) make this configuration useful for applications such as: blood coagulation, cancer treatment, sterilization, and waste treatment. Moreover, this setup can be potentially used as a test bed for the in-depth understanding of plasma chemistry relevant to the aurora and comet tails using a laboratory setting.

## Introduction

Research on low temperature atmospheric pressure plasma jets (APPJs) has immensely grown over the last couple of decades due to its variety of applications in diverse fields. So far, major implications of APPJs have been reported in the field of plasma medicine^1^, material processing^2^, and water purification^3^. Production of various reactive species such as reactive nitrogen species (NO and ${\text {NO}}_2$) and reactive oxygen species (O, OH and ${\hbox {O}}_{2}^-$) make plasma jets useful for these applications^4^.

Another interesting feature of atmospheric pressure plasma jets that hasn’t been investigated in detail is auroral physics. Given the abundance of ${\text {O}}_{2}$ in ambient air and the process of APPJs, atomic oxygen produces the emission of the green “auroral” line; a brilliant green line of wavelength 557.7 nm. The source of this line is the transition between metastable states of oxygen $\hbox {O}({}^1\hbox {S}) \, \rightarrow \, \hbox {O}({}^1\hbox {D})$.

Auroral physics was majorly studied by creating artificial emission of the 557.7 nm line in the ionosphere^5^. At higher pressure ($> 10$ Torr), an ${\text {O}}_2$ deficient environment is required to sustain the O($^1$S) line^6^. This is the reason why the green line is typically observed in altitudes ($>100 \, \hbox {km}$) where lighter atoms (such as O) are more abundant than the heavy oxygen molecule. Therefore, an open atmosphere often makes it difficult to observe such lines due to high collision rates and strong abundance of quenching molecules such as ${\text {O}}_{2}$ and ${\text {N}}_{2}$.

The chemical composition of Earth’s atmosphere contains ${\text {CO}}_{2}$, ${\text {O}}_{2}$, ${\text {N}}_{2}$, ${\text {H}}_{2} \hbox {O}$, Ar, and other trace gases. Other solar system objects (Mars, comets, etc.) contain the same ingredients although in highly different concentrations. The physical processes such as dissociation, excitation, and recombination are common to the aforementioned objects as well as atmospheric plasmas. In particular, a common pathway for O($^1$S) production originates from ${\text {H}}_{2} \hbox {O}$, a prevalent ingredient in comets and on Earth. Other production routes involve ${\text {CO}}_{2}$ and CO. The ratio of the O($^1$S) to O($^1$D) emission serves as an indicator of parent species on comets such as C/1996 B2 Hyakutake^7^.

Plasma chemistry involving the 557 nm line is relevant for the study of auroral processes and spectral calibration. The ratio of $[\hbox {O}({}^1 \hbox {D}) - \hbox {O}({}^3 \hbox {P})]/[\hbox {O}({}^1 \hbox {S}) - \hbox {O}({}^1 \hbox {D})]= \lambda _{630}/\lambda _{557}$ intensity is also important for model calibration^8^. The existence, or lack thereof, of 297 nm light from the relaxation of O($^1$S)–O($^3$P) is relevant for upper atmospheric studies on Mars, even though the chemical makeup of Mars is different from Earth. The I(557) nm and I(297) nm forbidden emissions originate from the same upper state, O($^{1}$S), so their intensity ratio should be the ratio of their transition probabilities. According to NIST^9^, this value is 16.7, but observations in the aurora have yielded different results^10^.

As of now, only a few researchers have tried to artificially form the green line at atmospheric pressure using mixtures of ${\text {N}}_2/{\text {O}}_2$ in either dielectric barrier discharge (DBD) or in microwave induced argon plasma jets where the green line was reported to be from the emission of the $\hbox {O}({}^1\hbox {S}) {\text {N}}_2$ excimer^11^. However, the experiments in DBD discharges using ${\text {N}}_2/{\text {O}}_2$ mixtures only measured weak emission from the O($^1$S) compared to NO and ${\text {N}}_2$. Panousis et al.^12^ showed relatively strong emission from the $\hbox {O}({}^1 \hbox {S}) {\text {N}}_2$ excimer in a DBD flowing ${\text {N}}_2$ afterglow within a quartz tube. However, the green line was not clearly visible in the photograph. Gherardi et al.^13^ measured the $\hbox {O}({}^1 \hbox {S}) {\text {N}}_2$ excimer using a parallel plate DBD inside a vacuum chamber filled to atmospheric pressure with ${\text {N}}_{2}$. On the other hand, in a microwave induced plasma jet, stability of the plasma was always an issue and the plasma streamers often form around the core resulting in heating and melting of the tube. Therefore, the system required an additional surrounding ${\text {N}}_2$ gas, to stabilize the discharge. As a side effect, the shielding gas molecules interacted with the core plasma and generated energetic nitrogen particles. Furthermore, the formation of auroral lines was mainly dependent on ${\text {N}}_2$ concentration and microwave power. As a result, these lines were observed only at higher ${\text {N}}_2$ flow rate and at lower power. Moreover, the plasma length was 1 cm which may not be very useful from the perspective of applications^11,14^.

In this paper, we present the first investigation on the novel phenomenon of auroral green line emission in an APPJ using a kHz frequency plasma source. We use pure argon (99.999%) as a working gas for the stable plasma generation without the need for secondary gases such as ${\text {N}}_2$, ${\text {O}}_2$ or ${\text {N}}_2/{\text {O}}_2$ admixture. The level of ${\text {N}}_{2}$ and ${\text {O}}_{2}$ impurities, on the order of 1–5 ppm, in the argon gas are not meaningful enough to affect the plasma generation in this manner. Using optical emission spectroscopy (OES) and imaging techniques we explored the behavior of green plasma jet in the ambient air for a wide range of parameters (gas flow rate, discharge voltage and electrode gap) considering a variety of medical and industrial applications. The chemical reactions of O($^{1}$S) production in the plasma have been established and the density of the O($^1$S) metastable has been estimated using the kinetic reactions. We measured the gas temperature to ensure that the plasma is useful for biomedical application. We further tested the efficacy of the plasma jet for industrial applications by treating a solution of methylene blue dye.

**Figure 1:**
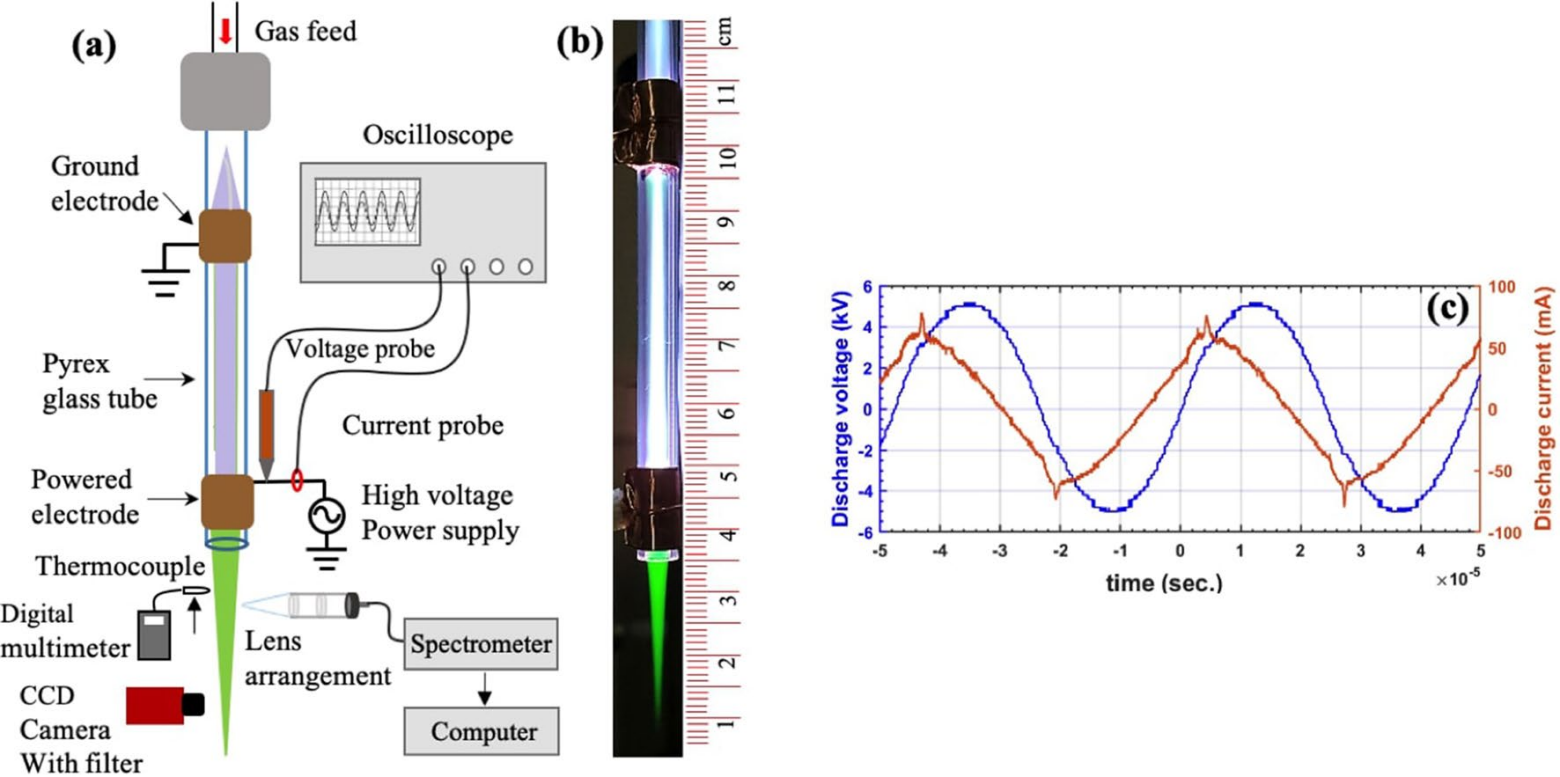
(**a**) Schematic diagram of the experimental setup. Diagnostics include optical emission spectroscopy, CCD camera, discharge voltage and current probes and multimeter with thermocouple. (**b**) Typical photograph of the APPJ. (**c**) Typical discharge voltage ($V_d$) and current ($I_d$) profiles of the plasma jet formed at $f = 21 \, \hbox {kHz}$, $V_d = 10.2 \, \hbox {kV}$ and gas flow rate $Q_{Ar} =6 \, \hbox {sL/min}$.

## Results and discussion

### Plasma jet characteristics

**Figure 2:**
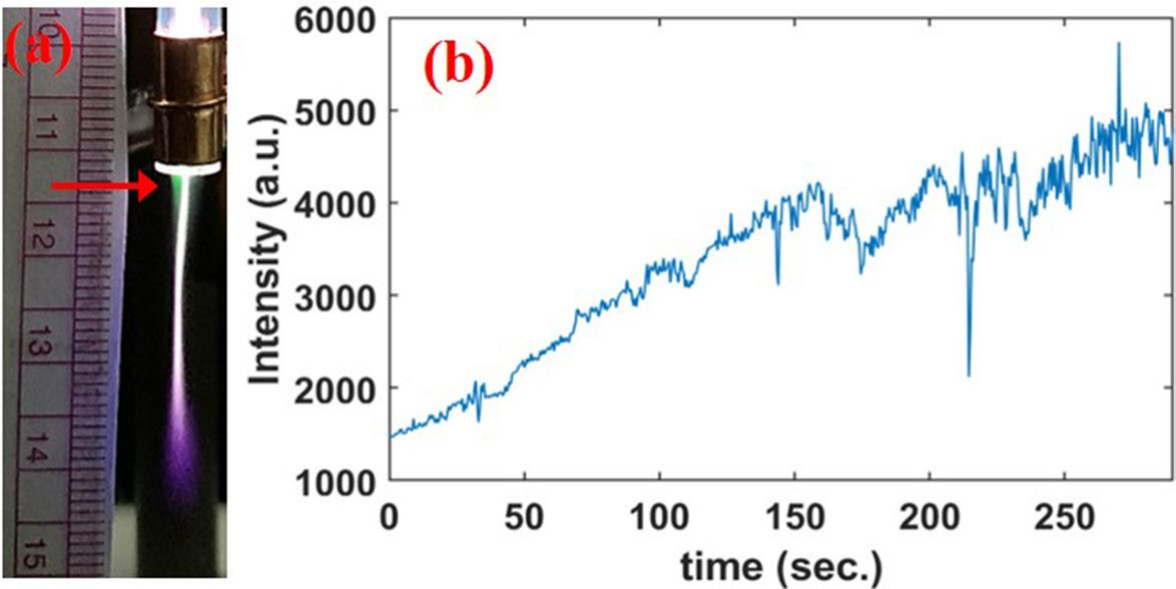
(**a**) Photograph of argon plasma jet in the ambient air ($V_d = 10.8 \, \hbox {kV}$, $f = 21 \, \hbox {kHz}$, ${\text {Q}}_{Ar} = 6 \, \hbox {sL/min}$, electrode gap $= 40 \, \hbox {mm}$) after initial plasma creation. (**b**) Time evolution of 557.7 nm emission line.

**Figure 3:**
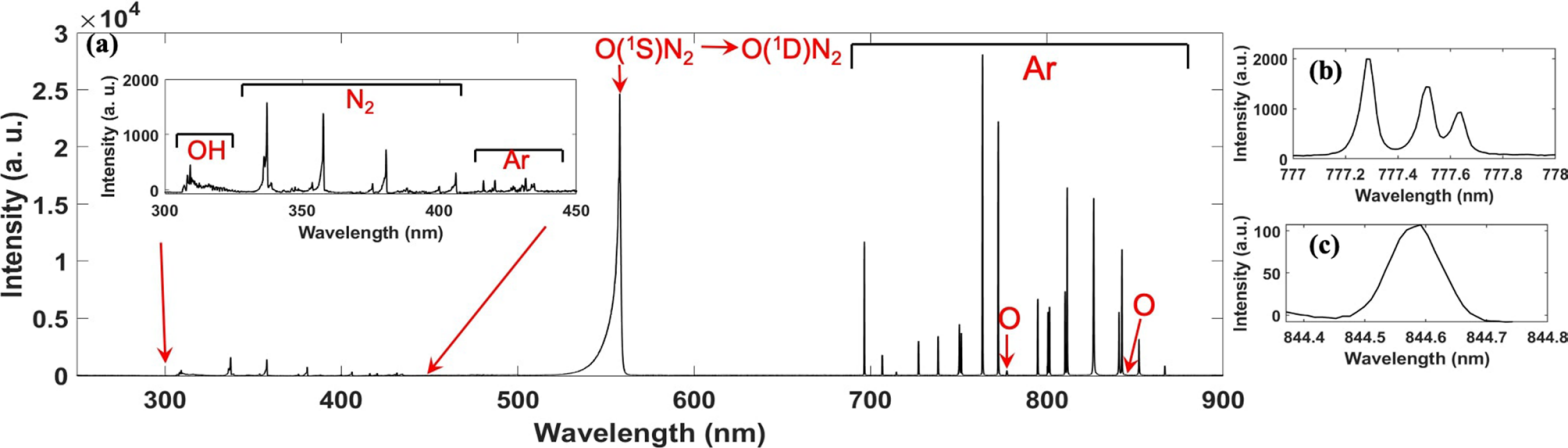
(**a**) Emission spectra from 250 nm to 900 nm observed in the APPJ ($V_d = 10.8\, \hbox {kV}$, $f = 21\, \hbox {kHz}$, ${\text {Q}}_{Ar} = 6\, \hbox {sL/min}$, electrode gap $= 40\, \hbox {mm}$). (**b**) High resolution spectra of the oxygen triplet at 777 nm. (**c**) High resolution spectra of oxygen at 844.6 nm.

Figure 1 shows a schematic of the plasma jet, while the details are discussed later, along with a typical photograph and voltage and current profiles. During the operation of the APPJ at optimum gas flow (6 sL/min) and discharge voltage (10.8 kV), initially a core argon plasma plume is formed in the ambient air as shown in Fig. 2a. The plasma jet is approximately 3 cm in length where we do not see any significant green light whereas it appeared closer to the nozzle as indicated by the arrow in the photograph. The green light grows over time and after 160 s the entire plasma becomes green. The emission intensity of the produced green line in the plasma jet is strong enough that it can be seen by the naked eye and can be recorded from a simple digital camera as shown in Fig. 1b. The intensity of an emission line is proportional to the population of the corresponding excited species. Therefore, we recorded the evolution of the 557.7 nm line with time (0.5 s resolution) as shown in Fig. 2b at a measurement location 5 mm below the nozzle. The intensity of 557.7 nm emission increases almost linearly and then saturates after 160 s. At this time, the green plasma is fully formed as shown in Fig. 1b. The green emission continues as long as we operate the plasma. The temporal evolution of the green plasma plume is explained by multiple factors. The green plasma is observed upon initial discharge of the plasma jet only at the nozzle. Any impurities, such as ${\text {H}}_{2} \hbox {O}$, in the gas line and glass tube are subsequently removed with further plasma generation. Since our measurement location is in the middle of the plasma plume, the dissociation of oxygen to envelop the entire plume takes some time. Since oxygen (${\text {O}}_{2}$) is a main quencher of the O($^{1}$S) state, it is gradually transformed into atomic oxygen throughout the plasma plume.

Figure 3a shows the emission spectra ranging from 250–900 $\hbox {nm}$ acquired from the after discharge region of the plasma jet. The spectrum shows strong emissions from the green line $[\hbox {O}({}^{1} \hbox {S}) \, \rightarrow \, \hbox {O}({}^{1} \hbox {D})]$ at 557.7 nm and Ar I emission from 696 nm to 866.8 nm marked by the horizontal bar in the figure. The plasma jet is characterized by the highly intense green line at 557.7 nm. The commonly observed O($^1$D) line at 630 nm in terrestrial auroras is not observed in this case. Thus the APPJ is able to generate copious amounts of O($^1$S) atoms to exhibit a highly visible green color. This supports the previous observation in laboratory based auroral line experiments^11,15^. Other atomic oxygen emission lines are also observed at 777 nm and 844 nm (Fig. 3c) though these lines are very weak compared to the 557.7 nm emission. Using a high resolution grating (2400 l/mm), we resolve all three transitions: 777.4 nm, 777.6 nm and 777.7 nm as presented in Fig. 3b. A few other weak emission lines are observed between 300–450 nm. An enhanced view is presented in the inset of Fig. 3a. This emission spectra consists of the OH band (306–312 nm) and various ${\text {N}}_{2}$ bands, most prominently the ${\text {N}}_{2}$ second positive system ranging from 337–380 nm. The ${\text {N}}_{2}$ second positive system is used to calculate the vibrational temperature ($T_{vib}$) of the plasma jet system. The gas temperature is measured with an insulated thermocouple and by using the Boltzmann plot method yielding 303 K and $300\pm 10 \, \hbox {K}$ respectively. The three lines of the ${\text {N}}_{2}$ second positive system (380 nm, 375 nm, and 370 nm) are used in the Boltzmann plot method^16^. This confirms that the plasma is non-thermal and room temperature which is required for many biomedical applications.

**Figure 4:**
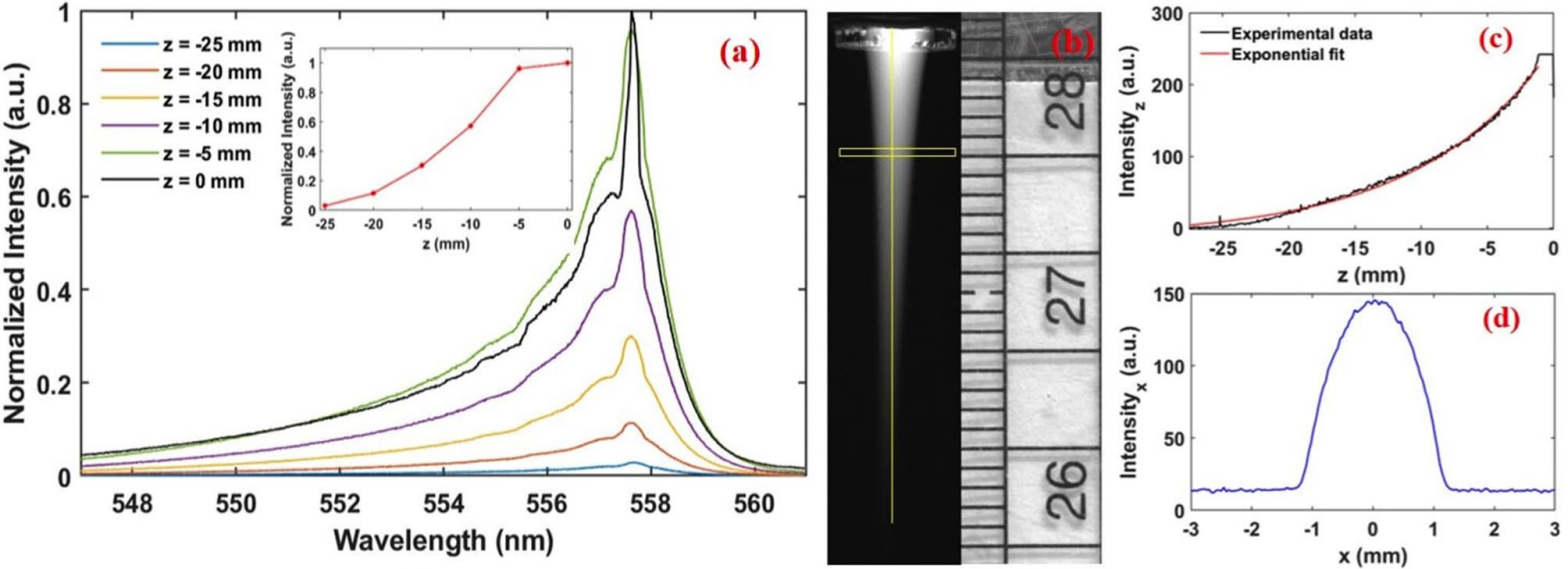
(**a**) Normalized emission spectra of O($^1$S) at different locations within the plasma plume. Inset shows maximum intensity variation with respect to vertical position with $z=0\, \hbox {mm}$ corresponding to the glass nozzle. (**b**) Typical image of the plasma jet taken from CCD camera. (**c**) The pixel intensity corresponding to the yellow vertical line shown in (**b**). (**d**) The pixel intensity extracted from the region between the horizontal box shown in (**b**).

We further measure the radial and axial spreading of the green emission in the ambient air by imaging and highly resolved OES techniques. This yields a measure of plasma volume which is an important parameter for a variety of applications^17^. Small size of APPJs often limit their use to small scale processes and materials treatment^18^. On the other hand, a system with bigger plasma volume is more efficient for general applications. Figure 4a presents the well resolved emission spectra of the 557.7 nm line at different vertical locations within the after discharge region. The intensity variation (in normalized units) is plotted as a function of vertical position in the inset of the Fig. 4a. The glass nozzle position or the beginning of the plasma plume, is defined as $z=0\, \hbox {mm}$ and the spectra is taken at every 5 mm. It is clear from the figure that the relative intensity decreases exponentially with increasing distance from the nozzle. However, a significant intensity is measured up to 25 mm from the nozzle. This indicates that the plasma jet length is at least 25 mm. For further verification we have also calculated the plasma length from the CCD image of the plume as shown in Fig. 4b. The yellow vertical line and a horizontal box in the figure shows the region of interest for the pixel intensity measurement along the axial (z) (see Fig. 4c) and radial direction (see Fig. 4d) respectively. It is found that the intensity decays exponentially with a typical rate of $0.1\pm 0.02\, {\hbox {mm}}^{-1}$. This result matches with the decay rate calculated from the spectral intensity variation shown in the inset of Fig. 4a. The length and width of the plasma (at $z = 5 \, \hbox {mm}$) is calculated as $25\pm 1\, \hbox {mm}$ and $2.5\pm 0.1\, \hbox {mm}$ respectively. The measured plasma dimensions are ideal to accomplish any atmospheric pressure plasma jet applications.

**Figure 5:**
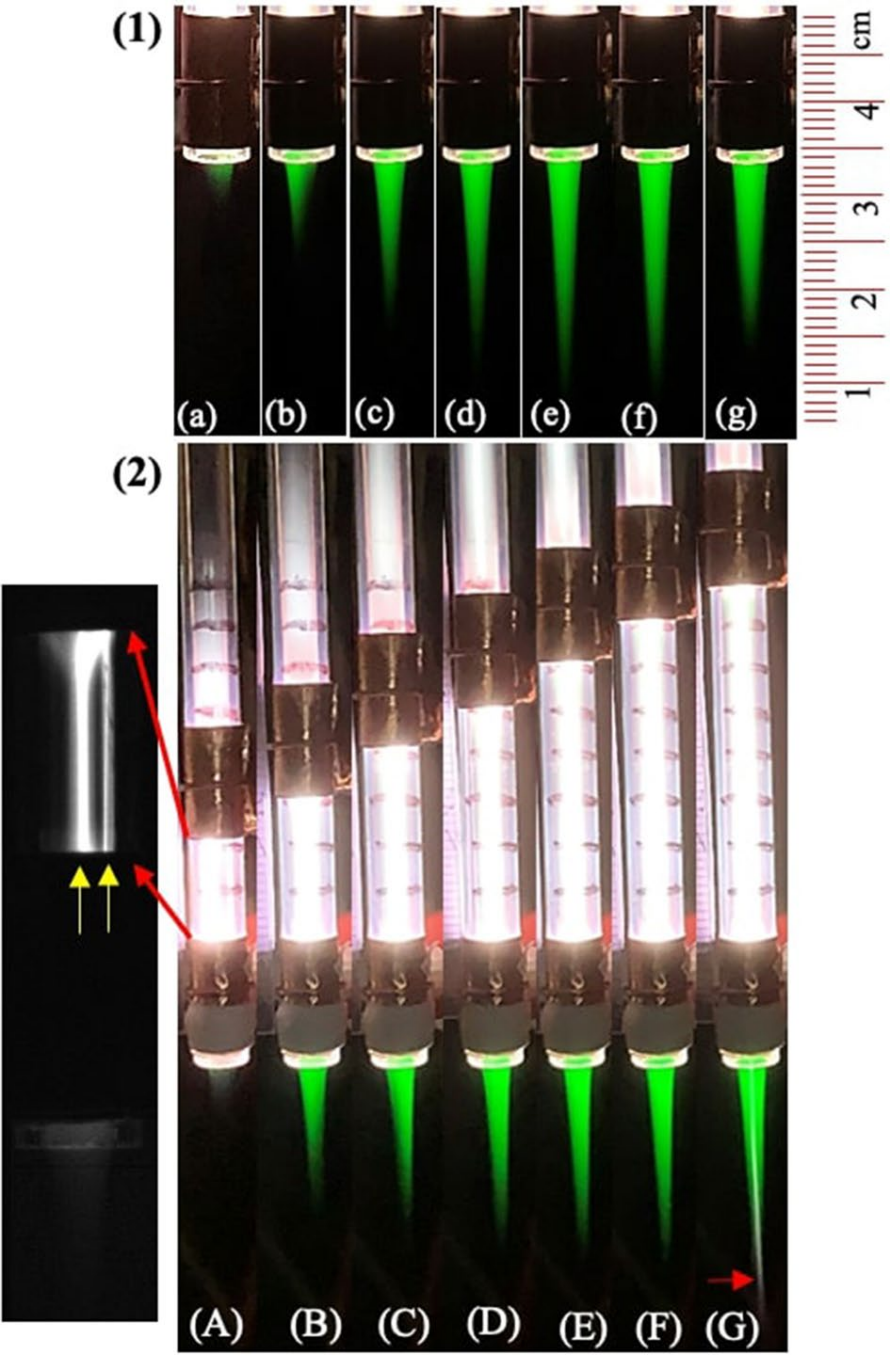
(1) Typical snapshots of the plasma jet at (**a**) 2 sL/min (**b**) 3 sL/min (**c**) 4 sL/min (**d**) 5 sL/min (**e**) 6 sL/min (**f**) 7 sL/min and (**g**) 8 sL/min. (2) Photograph of the APPJ at electrode gap (**A**) 10 mm, (**B**) 15 mm, (**C**) 20 mm, (**D**) 25 mm, (**E**) 30 mm, (**F**) 35 mm, (**G**) 40 mm. The b/w image represents the filaments formed between the electrodes for a gap of 10 mm.

**Figure 6:**
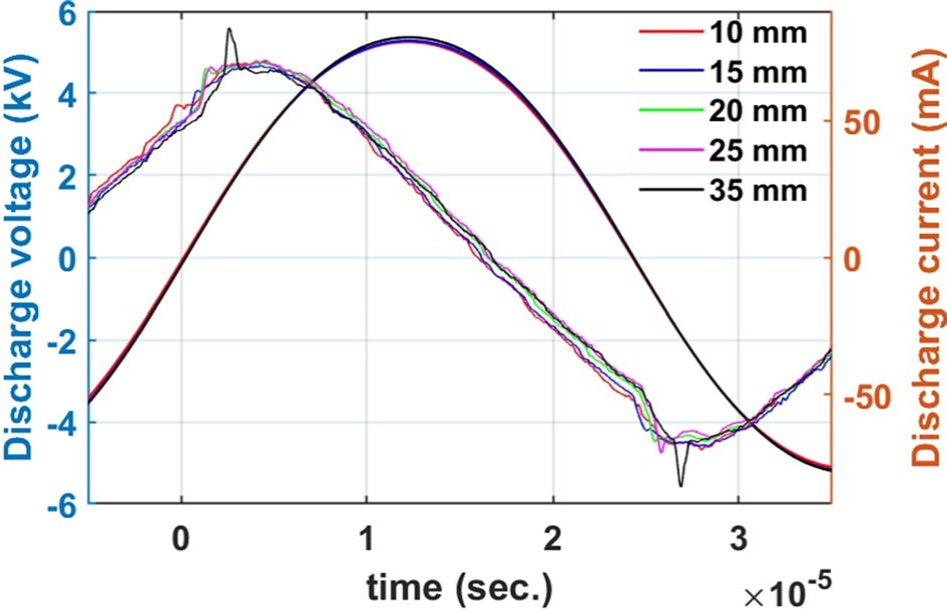
Discharge characteristics of the plasma jet for different electrode gaps while keeping constant discharge voltage ($V_{d}=10.8 \, \hbox {kV}$), frequency ($f = 21\, \hbox {kHz}$) and gas flow rate ($Q_{Ar}=6 \, \hbox {sL/min}$).

**Figure 7:**
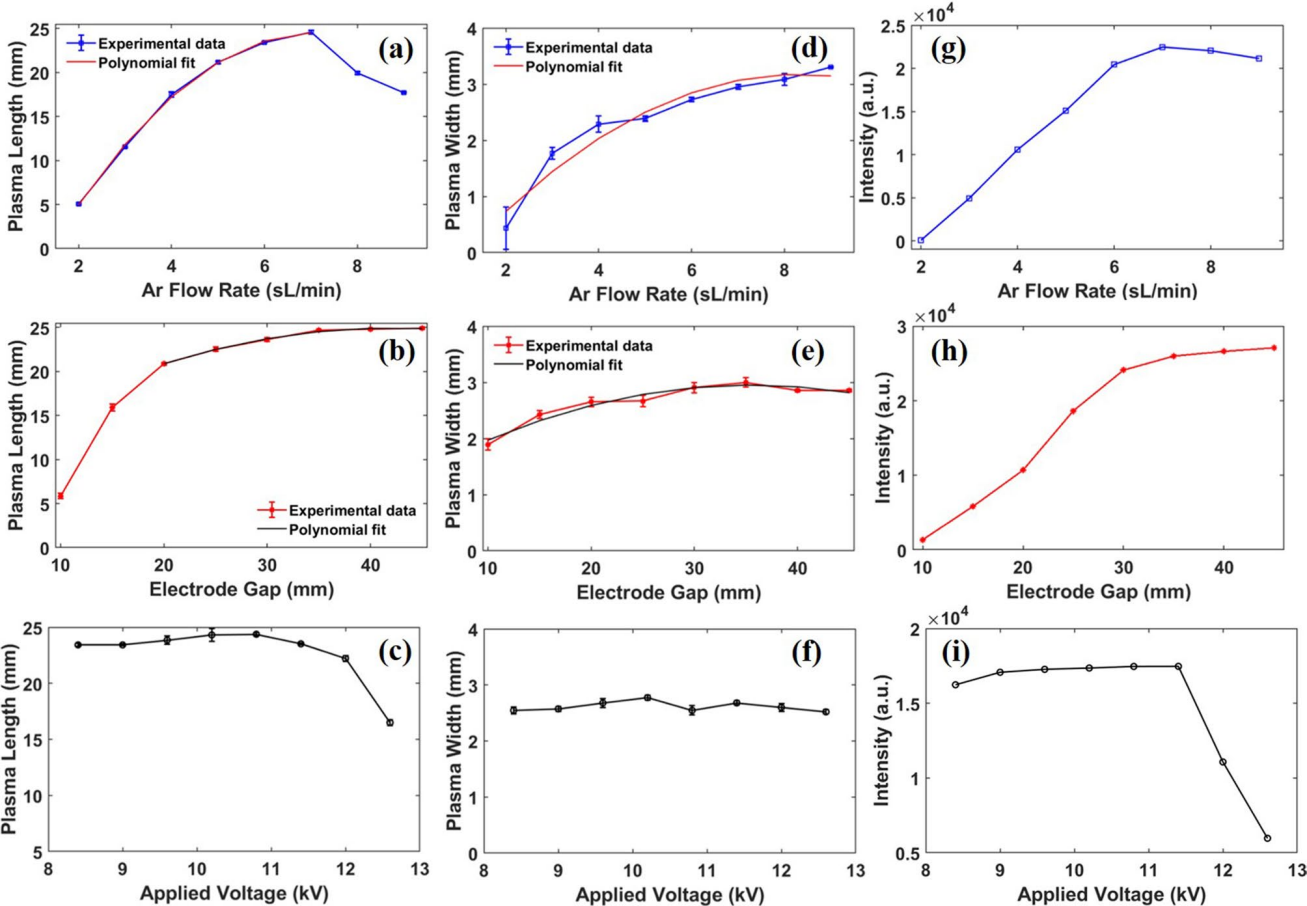
APPJ length, width, and 557.7 nm intensity variations with respect to the operating parameters. (**a**), (**d**), and (**g**) show the variation with respect to gas flow rate. (**b**), (**e**), and (**h**) show the variation with respect to electrode gap. (**c**), (**f**), and (**i**) show the variation with respect to applied voltage.

In order to understand the plasma dynamics in more detail, we further characterized the plasma behavior in terms of plasma length, width and intensity with respect to the argon flow rate and electric field, either by changing the electrode gap or by applied voltage. Figure 5(1) shows typical snapshots of the plasma jet while varying the argon flow rate at a fixed electrode gap of 40 mm. Figure 5(2) shows the plasma jet while changing the electrode gap at a fixed flow rate of $Q_{Ar}=6\, \hbox {sL/min}$. In all cases, the entire plasma plume is always dominated by the 557.7 nm emission regardless of argon flow rate and electrode gap albeit the overall plasma characteristics show dependence on the operating parameters. The plasma jet is found to be more sensitive to the gas flow rate as reflected from the photographs which is similar to previous work^19^. However at relatively larger electrode gap (above 40 mm) a filament is formed in the middle of the plume with purple color visible at the tip as pointed out by the arrow in Fig. 5(2)G. Overall the plasma plume for all cases seems more diffusive rather than the conventional filamentary Ar plasma jet^20^. This may be related to the excess singlet O formation^21^. In contrast, for the smaller inter electrode gap (at 10 mm) the plasma is more filamentary in the inter electrode region which is reflected from the CCD image in Fig. 5(2). At this small inter electrode gap the length of the plasma jet outside the nozzle is also small, which may be due to the nearby ground electrode which neutralizes the positive charges by the polarization charges induced in the region, thus terminating its further propagation^22,23^. Further, the increase in the inter electrode gap leads to increase in the plasma length in the ambient air which is related to the increase in the ionization as the increased length of the discharge region.

Moreover, the discharge voltage and current characteristics for variation of inter electrode gap while keeping constant discharge voltage ($V_{d} =10.8 \, \hbox {kV}$), frequency ($f = 21\, \hbox {kHz}$) and gas flow rate ($Q_{Ar} =6 \, \hbox {sL/min}$) is shown in Fig. 6. The variation in inter electrode gap leads to minor change in the discharge current characteristics whereas the voltage profile is unaffected. As we increase the inter electrode gap, the discharge current peaks shifts to right. This behaviour is associated to the increase in the discharge path which delays the discharge peak. A significant discharge is observed in both the half cycles for the electrode gap of 35 mm which remain the same for further increase in the electrode gap. Meanwhile, a small variation in the phase shift ($5^{\circ }$) is also noticed with the changing electrode gap attributed to the change in the impedance.

Figure 7 depicts the variation of the plasma jet (length, width and intensity) as a function of gas flow rate, electrode gap and discharge voltage. The parameters are calculated using OES and by analyzing the CCD images as similar to Fig. 4. The polynomial fitting in Fig. 7a shows a nonlinear variation in the plasma length as we increase the gas flow rate from 2–7 sL/min after which the plasma length begins to decrease. The intensity of the O($^{1}$S) emission (see Fig. 7g) follows a similar trend where it increases linearly up to 7 sL/min and decreases after that. However, the width of the plasma jet is continuously increasing with higher $Q_{Ar}$ as shown in Fig. 7d. All other operating parameters are kept constant ($V_d = 10.8\, \hbox {kV}$, $f = 21\, \hbox {kHz}$ and gap = 40 mm). We can understand the change in the plasma jet characteristics from the fluid dynamic calculations. As the flow rate is altered, the fluid regime is determined from the Reynolds number (Re) given by:

1 $$\begin{aligned} \text {Re}=\frac{4 \rho Q}{\mu \pi D} \end{aligned}$$

where $\rho$ is the density, *Q* is the flow rate, $\mu$ is the dynamic viscosity, and *D* is the inner tube diameter. The inner tube diameter is 4 mm, the dynamic viscosity is $2.23 \times 10^{-5} \, {\hbox {N s/m}}^{2}$ and the argon density is $1.66 \, {\hbox {kg/m}}^{3}$. Since the temperature (293 K) is assumed constant, $\rho$ and $\mu$ are also constant for all flow rates. Table 1 shows the Reynolds number versus the argon flow rate. It is generally accepted that for pipe flow, Re $<2000$ represents the laminar regime and Re $>4000$ is the turbulent regime^24^. For our case the plasma jet exhibits laminar mode up to 7 sL/min, while turbulent mode is produced with further increase in gas flow rate. The transition from laminar to a turbulent mode yields a significant decrease in the plasma jet length.

**Table 1:** Gas parameters and Reynolds number for various flow rates of argon.

Flow (sL/min)	Q (${\hbox {m}}^{3}/\hbox {s}$)	Re
2	$0.33\times 10^{-4}$	1007
3	$0.50\times 10^{-4}$	1510
4	$0.67\times 10^{-4}$	2014
5	$0.83\times 10^{-4}$	2517
6	$1.00\times 10^{-4}$	3021
7	$1.17\times 10^{-4}$	3524
8	$1.33\times 10^{-4}$	4028
9	$1.50\times 10^{-4}$	4531

Changing the electrode gap creates a similar effect on the plasma jet characteristics. Figure 7b, e and h shows the variation in plasma jet characteristics with a changing electrode gap at constant frequency, voltage and gas flow rate ($f = 21\, \hbox {kHz}$, $V_d = 10.8\, \hbox {kV}$ and $Q_{Ar} = 6\, \hbox {sL/min}$). The length and O($^{1}$S) intensity both increase with increasing electrode gap up to 30 mm. There is no major change observed in the width of the plasma jet (Fig. 7e) except a small decrease when the electrode gap is below 15 mm. As discussed earlier, the plasma regime changes when the electrode gap is less than 15 mm. The smallest dimension plasma jet is found to be 5 mm in length and 2 mm width when the electrode gap is 10 mm.

The plasma jet is further characterized by altering the discharge voltage from 8.4 to 12.6 kV while keeping the other operating parameters constant (electrode gap = 40 mm, $Q_{Ar} = 6\, \hbox {sL/min}$, and $f = 21\, \hbox {kHz}$). The plasma is formed for discharge voltages above 8 kV. The maximum voltage of the power supply is 13 kV. As indicated from Fig. 7c and i the plasma jet length and O($^{1}$S) intensity are approximately constant up to 11.6 kV after which they begin to decrease. Above 11.6 kV we observed the formation of filaments between the electrodes, similar to the case of an electrode gap of 10 mm. This defines a threshold in the electric field above which the homogeneous glow discharge mode becomes filamentary, resulting in a significant change in the plasma jet. The plasma jet width is unaffected by the discharge voltage as shown in Fig. 7f, similar to the effect when the electrode gap is altered.

### Auroral line formation

This section is dedicated to gain more insight of the formation of auroral green emission in a pure argon APPJ. We use 99.999% pure argon gas with impurity levels of 1 ppm and 5 ppm for ${\text {O}}_{2}$ and ${\text {N}}_{2}$, respectively. Based on past studies^11,15^, such small levels of impurities, even in lower grade argon gas, would not be enough to affect the 557.7 nm emission shown here. Quenching molecules such as ${\text {O}}_{2}$ and ${\text {N}}_{2}$ are much higher in ambient air. Previous work that observed O($^{1}$S) emission consistently used nitrogen as the working gas^11^. Small amounts of ${\text {O}}_{2}$ mixture were always required for O($^{1}$S) emission^11^. In those systems, the O($^{1}$S) emission was not observed throughout the entire plasma plume. If any emission was observed, it was weak and mainly dependent on nitrogen gas concentration. Therefore, those systems created mostly reactive nitrogen species. In contrast, our system produces reactive oxygen and nitrogen species which have numerous biological benefits. Based on our experimental procedure to create the plasma jet and the most prominent emission lines observed in the after discharge region, the atomic oxygen is most likely created by a quenching reactions of argon metastables with oxygen molecules

2 $$\begin{aligned} \text {Ar}^{*} + \text {O}_{2} \rightarrow 2\text {O}+\text {Ar} \end{aligned}$$

with $\hbox {k} = 5.8\times 10^{-11} \text {cm}^3 \text {s}^{-1}$^25^. The argon emission that results in the $1s_{5}$ metastable state clearly shows us that this state is highly populated. Studies of the argon $1s_{5}$ metastable density in atmospheric plasma jets yield a density of $10^{10}-10^{13} \, {\hbox {cm}}^{-3}$^26–28^. Once the atomic oxygen is created, the O($^{1}$S) state is formed through a variety of reaction pathways. The reaction with nitrogen

3 $$\begin{aligned} \text {O}(^{3}\text {P}) + \text {N}_{2}(\text {A}) \rightarrow \text {O}(^{1}\text {S}) + \text {N}_{2} \end{aligned}$$

is considered to be the source for the O($^{1}$S) according to multiple studies^11,13,29^. That is not the case in our work which sees no evidence of the ${\text {N}}_{2}$(A) state through emission of the ${\text {N}}_{2}$ 1st positive system. On the other hand, the O($^{1}$S) state is only 4.19 eV above the ground state. Such a low lying level can easily be excited via electron impact,

4 $$\begin{aligned} \text {e} + \text {O} \rightarrow \text {O}(^{1}\text {S}) + \text {e} \end{aligned}$$

rather than a more complicated sequence. There are various possible loss mechanisms of O($^{1}$S) including: collisional, radiative and electron impact. However, radiative loss is the dominant loss mechanism through which O($^{1}$S) decays as $\text {O}(^{1}\text {S})\rightarrow \text {O}(^{1}\text {D}) + h\nu (557.7 \text{ nm})$. In a nitrogen dominated environment, O($^{1}$S) reacts with the nitrogen molecule to form the $\hbox {O}({}^{1}\hbox {S}) {\text {N}}_2$ excimer which subsequently relaxes through the reverse reaction,

5 $$\begin{aligned} \text {O}(^{1}\text {S}) + \text {N}_{2} + \text {N}_{2} \underset{k_d, k_{-d}}{\longleftrightarrow } \text {O}(^{1}\text {S})\text {N}_{2} + \text {N}_{2} \end{aligned}$$

where $k_d = 2\times 10^{-36} \text {cm}^6\text {s}^{-1}$ and $k_{-d} = 5\times 10^{-12} \text {cm}^3\text {s}^{-1}$ are the rate constant for forward and reverse reaction respectively^11^. The $\hbox {O}({}^{1} \hbox {S}) {\text {N}}_2$ de-excites to the $\hbox {O}({}^{1} \hbox {D}) {\text {N}}_2$ state with a release of a green photon of 557.7 nm at a rate constant of $k = 1\times 10^7 \text {s}^{-1}$. The excimer emits a molecular band resulting in the characteristic skewed line shape observed in our experiment. The only oxygen lines that we observe besides the O($^{1}$S) at 557.7 nm is the triplet at 777 nm and the triplet at 844 nm as shown in Fig. 3b and c. It appears that most of the atomic oxygen finds its way to the O($^{1}$S) state rather than anywhere else. Both the O($^{1}$S) and O($^{1}$D) are considered metastables, even though they can radiatively decay, because of their long lifetimes (0.8 s and 110 s respectively)^30,31^. Notably, there is no observed emission from the O($^{1}$D) to the ground state O($^{3}$P) at 630 nm. Given the lifetime of the O($^{1}$D), it is likely that the state is collisionally quenched by ${\text {O}}_{2}$ or ${\text {N}}_{2}$ or it is re-excited to the O($^{1}$S). Both the O($^{1}$S) and O($^{1}$D) states are important species because they can react with water to form OH;

6 $$\begin{aligned} \text {O}(^{1}\text {S}) + \text {H}_{2}\text {O} \rightarrow \text {OH} + \text {OH} \end{aligned}$$

with $k = 3.0 \times 10^{-11} \, {\hbox {cm}}^{3} \, {\hbox {s}}^{-1}$^32^. In our experiment the collisional loss mechanism of O($^{1}$S) is the main source of OH. Plasma jets primarily made with ${\text {N}}_{2}$ lack this ability as they instead produce NO^15,29,33^.

The visually intense green emission and broad spectra have already concluded the strong abundance of O($^{1}$S) in our APPJ system qualitatively. Now we calculate the concentration of O($^{1}$S) in atmospheric pressure argon plasma jet quantitatively. In order to do so we follow the method by Pointu et al.^15^ where the calculation is based on production and loss rate of the radiative states. In our case the nitrogen second positive system is the comparative radiative state besides $\hbox {O}({}^{1} \hbox {S}) {\text {N}}_2$. By comparing the emission intensity of excited ${\text {N}}_2$ and $\hbox {O}({}^{1} \hbox {S}) {\text {N}}_2 \, \rightarrow \, \hbox {O}({}^{1} \hbox {D}) {\text {N}}_2$ we can calculate the density of the O($^{1}$S) metastable since the main loss term of the $\hbox {O}({}^{1} \hbox {S}) {\text {N}}_2$ excimer is the inverse reaction, with a rate coefficient $k_{-d}$. Therefore the pseudo-stationary density of $\hbox {O}({}^{1} \hbox {S}) {\text {N}}_{2}$ is given by the following equation:

7 $$\begin{aligned} \left[ \text {O}(^1\text {S})\text {N}_2\right] = \frac{\left[ \text {O}(^1\text {S})\right] \left[ \text {N}_2 \right] k_d}{k_{-d}} \end{aligned}$$

The excitation of ground state nitrogen is given by

8 $$\begin{aligned} \text {Ar}^{*} + \text {N}_{2} \underset{k_a}{\rightarrow } \text {N}_2(\text {C})+\text {Ar} \end{aligned}$$

while the loss of excited nitrogen is mainly due to radiative de-excitation and ${\text {N}}_2$ quenching as given by

9 $$\begin{aligned} \text {N}_2(\text {C})\rightarrow \text {N}_2(\text {B},0)+\text {photon (337.1 nm)} \end{aligned}$$

10 $$\begin{aligned} \text {N}_2(\text {C}) + \text {N}_{2} \underset{k_e}{\rightarrow } \text {N}_2(\text {B},0)+\text {N}_2 \end{aligned}$$

where $k_a = 3.0\times 10^{-11}~\text {cm}^3\text {s}^{-1}$ and $k_e = 1.1\times 10^{-11}~\text {cm}^3\text {s}^{-1}$^27^. The pseudo-stationary state density of ${\text {N}}_2$(C) is given as

11 $$\begin{aligned} \left[ \text {N}_2(\text {C})\right] =\frac{\left[ \text {Ar}^*\right] \left[ \text {N}_2\right] k_a}{\left[ \text {Ar}\right] k_e}. \end{aligned}$$

By comparing the emission intensity of ${\text {N}}_2$ at 337.1 nm and the O($^1$S) emission, the density of O($^1$S) is given as

12 $$\begin{aligned} \left[ \text {O}(^1\text {S})\right] = \frac{\left[ \text {Ar}^*\right] }{\left[ \text {Ar} \right] }\left\{ \frac{k_a k_{-d} A_{(337.1\,\mathrm{nm})}}{k_e k_d A_{(557.7\, \mathrm{nm})}}\right\} \frac{I_{(557.7\,\mathrm{nm})}}{I_{(337.1 nm)}}{\frac{c(\lambda _{337.1\,\mathrm{nm}})}{c(\lambda _{557.7\,\mathrm{nm}})}} \end{aligned}$$

where, $I_{(337.1\,\mathrm{nm})}$ and $I_{(557.7\,\mathrm{nm})}$ are the intensity of 337.1 nm and 557.7 nm respectively. ${\text {A}}_{(337.1\,\mathrm{nm})} = \hbox {A}({\text {N}}_2 (\hbox {C},0)\hbox {-} {\text {N}}_2 (\hbox {B},0)) = 1.21 \times 10^7 \text {s}^{-1}$ and ${\text {A}}_{(557.7nm)} = \hbox {A}({\text {N}}_2 \hbox {O}({}^1 \hbox {S}) \, \rightarrow \, {\text {N}}_2 \hbox {O}({}^1 \hbox {D})) = 1 \times 10^7 \text {s}^{-1}$ are the the radiative frequencies of the two considered transitions^11,27^. At 5 mm below the nozzle the peak intensity ratio of 557.7 nm to 337.1 nm is measured as 13. Since these two wavelengths are far apart, $c(\lambda _{337.1\,\mathrm{nm}})$ and $c(\lambda _{557.7\,\mathrm{nm}})$ are the spectral response of the spectrometer at 337.1 nm and 557.7 nm respectively. The spectral response was determined by using a calibrated National Institute of Standards and Technology mercury lamp. The ratio of the spectral response term in Eq. (12) was determined to be 0.461. For an argon pressure of 2 bar (measured in the gas line) the neutral density is calculated as $4.57\times 10^{19}~\text {cm}^{-3}$ assuming room temperature. Using these values in Eq. (12) and for $[{\hbox {Ar}}^*] = 1.4\times 10^{10}~\text {cm}^{-3}$ (taken from past studies^26,27^) the concentration of O($^{1}$S) is deduced as $1.4\times 10^{16}~\text {cm}^{-3}$ which is in the range of atomic oxygen calculated in APPJs. This indicates that most of the atomic oxygen is comprised of O($^{1}$S) which is also seen in Fig. 3a. Therefore, our plasma jet system is a very efficient laboratory device for O($^{1}$S) production.

**Figure 8:**
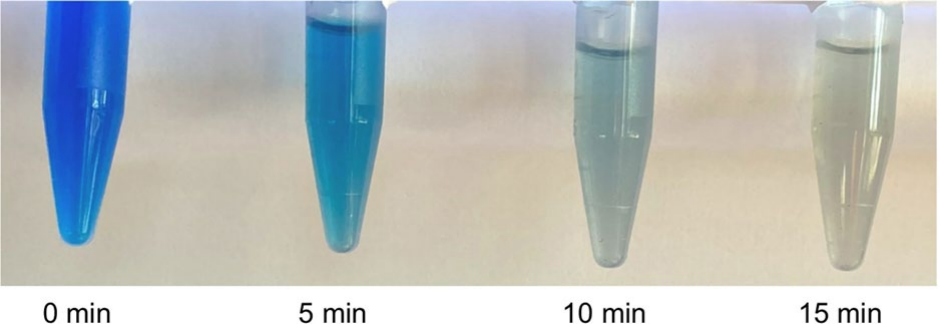
Photos showing the degradation of the methylene blue dye solution treated by the plasma jet every five minutes. The plasma jet parameters were $f = 21\, \hbox {kHz}$, $V_d = 10.2\, \hbox {kV}$ and gas flow rate $Q_{Ar}=6 \, \hbox {sL/min}$.

### Applications of O($^{1}$S)

The APPJ discussed here is also relevant for a variety of other applications which we will briefly mention. The strong abundance of reactive oxygen species make this system ideal for the applications such as cancer treatment, wound healing and antibiotic treatment^34, 35^. In particular, the $\hbox {O}({}^{1}\hbox {S}) {\text {N}}_{2}$ excimer was found to be the most efficient in sterilizing *Bacillus stearothermophilus* spores in spite of the low concentration^15^. The O($^{1}$S) emission in our case is much higher than any other species therefore, it can enhance the efficacy of sterilization. A high O($^{1}$S) concentration is useful to accelerate the biocidal effect. Also, the production of atomic oxygen makes this system suitable for blood coagulation^36^. Recent research involving atomic oxygen for plasma medicine applications has focused on redox signalling^37,38^, the response of a biological system to changes in the level of a particular reactive species. Oxygen is also critically important for cancer treatment. Plasma induced apoptosis for leukemia cells was found to be dependent on the feed gas and oxygen was the key ingredient^39^. To further determine the plasma jet effectiveness for applications, we treated a solution of methylene blue dye. After 15 min of plasma jet treatment, the dye was completely degraded and the solution was clear. Visual confirmation is provided in Fig. 8. The significant decomposition confirms that this system is also suitable for water purification. Undoubtedly, new applications of plasma jets producing oxygen will be realized in the near future.

## Conclusion

We have presented a kHz atmosphere pressure plasma jet that produces strong emission from the $\hbox {O}({}^{1} \hbox {S}) {\text {N}}_{2}$ excimer. Contrary to established understanding, the formation of the O($^{1}$S) state is not dependent on nitrogen as the working or shielding gas. The O($^{1}$S) emission persists for a range of operating parameters. The plasma jet was verified to be room temperature and safe to human touch. The production of a high concentration of atomic oxygen along with other reactive nitrogen and oxygen species and the successful degradation of the methylene blue dye confirms that this plasma jet has great applicability in plasma medicine, water purification and a variety of other applications. Moreover, given the rich particle species production of atmospheric plasmas, the system presented here is an ideal test bed for understanding plasma chemistry in the aurora, comet tails, and more. Our system reduces the major complexity of the plasma jet system and makes it very cost effective unlike previously used systems for artificial auroral emission.

## Methods

A complete schematic diagram of the experimental setup is shown in Fig. 1a. Argon (Ar) gas of 99.999% purity is introduced into the glass tube of outer diameter 6 mm and thickness 1 mm. Two copper electrodes of width 10 mm are arranged in a linear field configuration where the lower electrode (closer to the nozzle) is active and the upper electrode is grounded. High voltage, ranging from 8.4–12.6 $\hbox {kV}$, at a constant frequency $f = 21\, \hbox {kHz}$ is applied on the active electrode with a gas flow rate from 2–9 sL/min to excite the plasma. A typical photograph of the APPJ formed in the ambient air at a discharge voltage of 10.2 kV, $f = 21\, \hbox {kHz}$ and $Q_{Ar} = 6\, \hbox {sL/min}$ is shown in Fig. 1b. A voltage probe (P6015A, Tektronix, 75 MHz bandwidth) and current probe (TEK/TCP202) are used to measure the applied voltage ($V_{d}$) and discharge current ($I_{d}$) profiles respectively. Typical profiles of $V_{d}$ and $I_{d}$ are shown in Fig. 1c. A digital multimeter with a thermocouple is used to approximate the temperature of the plasma plume. The gas temperature measurement has been compared to those calculated from emission lines recorded using a high resolution ($\le 0.05 \, \hbox {nm}$) spectrometer (Princeton Instruments SpectraPro$^{\circledR}$ HRS-500). An optical arrangement (shown in Fig. 1a), featuring a plano-convex and meniscus lens with a multi-wavelength (200–1000 $\hbox {nm}$) collimator, is used to make a spot size $< 2 \, \hbox {mm}$ for a precise local measurement. The arrangement is attached to a 1 m long optical fiber which is aimed perpendicular to the plasma jet at a distance of approximately 10 cm from the edge of the plasma jet. The spectrometer integration time was set between 1–10 s to compensate for the varying signal from the plume. The image of the plasma jet is recorded using a high resolution CCD camera (Ximea MQ042MG-CM - XiQ - 4.2 MP, 90 fps) for the calculation of plasma volume. In order to measure the green light more accurately, a band pass filter of center wavelength $\lambda = 550 \pm 8 \, \hbox {nm}$, $\hbox {FWHM} = 40 \pm 8 \, \hbox {nm}$ is attached to the camera. The color photograph is taken by a digital camera with 12 M pixel resolution. The methylene blue solution was prepared by mixing 0.1 mL of 1% methylene blue dye dissolved in ethanol and 0.9 mL of deionized water. The solution was placed in a 1.5 mL centrifuge tube and placed below the plasma jet such that the plasma/liquid interface was at $z=-10\, \hbox {mm}$.
